# Long-term outcomes in patients with type 2 diabetes receiving glimepiride combined with liraglutide or rosiglitazone

**DOI:** 10.1186/1475-2840-8-12

**Published:** 2009-02-26

**Authors:** Sean D Sullivan, Rafael Alfonso-Cristancho, Chris Conner, Mette Hammer, Lawrence Blonde

**Affiliations:** 1 Pharmaceutical, Outcomes Research and Policy Program, University of Washington, Seattle, USA; 2 Novo Nordisk Inc, Princeton, NJ, USA; 3 Novo Nordisk A/S, Bagsvaerd, Denmark; 4 Ochsner Diabetes Clinical Research Unit, Department of Endocrinology, Ochsner Medical Center, New Orleans, LA, USA

## Abstract

**Background:**

Poor control of type 2 diabetes results in substantial long-term consequences. Studies of new diabetes treatments are rarely designed to assess mortality, complication rates and costs. We sought to estimate the long-term consequences of liraglutide and rosiglitazone both added to glimepiride.

**Methods:**

To estimate long-term clinical and economic consequences, we used the CORE diabetes model, a validated cohort model that uses epidemiologic data from long-term clinical trials to simulate morbidity, mortality and costs of diabetes. Clinical data were extracted from the LEAD-1 trial evaluating two doses (1.2 mg and 1.8 mg) of a once daily GLP-1 analog liraglutide, or rosiglitazone 4 mg, on a background of glimepiride in type 2 diabetes. CORE was calibrated to the LEAD-1 baseline patient characteristics. Survival, cumulative incidence of cardiovascular, ocular and renal events and healthcare costs were estimated over three periods: 10, 20 and 30 years.

**Results:**

In a hypothetical cohort of 5000 patients per treatment followed for 30 years, liraglutide 1.2 mg and 1.8 mg had higher survival rates compared to the group treated with rosiglitazone (15.0% and 16.0% vs. 12.6% after 30 years), and fewer cardiovascular, renal, and ocular events. Cardiovascular death rates after 30 years were 69.7%, 68.4% and 72.5%, for liraglutide 1.2 mg, 1.8 mg, and rosiglitazone, respectively. First and recurrent amputations were lower in the rosiglitazone group, probably due to a 'survival paradox' in the liraglutide arms (number of events: 565, 529, and 507, respectively). Overall cumulative costs per patient, were lower in both liraglutide groups compared to rosiglitazone (US$38,963, $39,239, and $40,401 for liraglutide 1.2 mg, 1.8 mg, and rosiglitazone, respectively), mainly driven by the costs of cardiovascular events in all groups.

**Conclusion:**

Using data from LEAD-1 and epidemiologic evidence from the CORE diabetes model, projected rates of mortality, diabetes complications and healthcare costs over the long term favor liraglutide plus glimepiride over rosiglitazone plus glimepiride.

**Trial registration:**

LEAD-1 NCT00318422; LEAD-2 NCT00318461; LEAD-3 NCT 00294723; LEAD-4 NCT00333151; LEAD-5 NCT00331851; LEAD-6 NCT00518882.

## Background

Type 2 diabetes is a chronic disease associated with insulin resistance and a progressive failure of the pancreatic beta cells. [1-3]. Type 2 diabetes is believed to account for about 90% of all cases of diabetes [4]. The American Diabetes Association (ADA) reported that, in the USA in 2007, 17.5 million people were diagnosed with diabetes. Estimates from the Centers for Disease Control and Prevention (CDC), which include persons with both diagnosed and undiagnosed diabetes, place the number of Americans with diabetes at 23.6 million [5]. The number of people with diagnosed diabetes is growing at a rate of 1 million per year [6], and is projected to reach over 48 million by 2050 [7]. The impact of diabetes on the US economy is alarming, with a total estimated cost of US$174 billion in 2007. A majority of the economic burden, $116 billion, can be attributed to expenditures for medical care [6]. A majority of these costs are for treatment of complications of the disease [8-11].

Large population-based studies have established that diabetes is associated with increased rates of cardiovascular morbidity and death [12-15]. Clinical trials have shown the benefits of intensive glucose lowering therapies to reduce the risk of microvascular disease [1], cardiovascular events and death [16,17], or the combined risk of micro- and macrovascular events [18], in diabetic patients. Diabetes-related complications greatly diminish patients' health-related quality of life [19-21]. More recently, new evidence suggest that intensive treatment and extreme reductions in HbA1c below 6.5%, may have no effect, or (in one study) even increase the rate of cardiovascular events and death in high risk patients with diabetes [18,22]. Thus, until this new evidence can be completely understood and supported by large longitudinal studies, it seem plausible that an intervention targeting reduction in glycemia levels to current guidelines, as well as improving concomitant risk factors, such as blood pressure, lipid levels and bodyweight might prevent and reduce the risk of micro- and macro-vascular complications. This intervention has recently been endorsed by a position statement of the American Diabetes Association and a scientific statement of the American College of Cardiology Foundation and the American Heart Association [23].

Liraglutide is a new once-daily human glucagon-like peptide (GLP)-1 analog. GLP-1 is a natural glucose-regulating peptide that enhances insulin secretion and reduces glucagon secretion, both in a glucose-dependent manner. Naturally occurring GLP-1 would require continuous infusion because of its short half-life, and so is impractical for routine therapeutic use; therefore, GLP-1 receptor agonists with an extended duration of action have been developed. The efficacy and safety of liraglutide treatment has been investigated both as monotherapy [24], and in combination with a number of currently approved therapies (metformin, sulfonylurea, thiazolidinediones) for type 2 diabetes in a large phase 3a trial program with extensive use of active comparators (the Liraglutide Effect and Action in Diabetes [LEAD 1–6] trial program) [25-30].

Our objective was to model the long-term outcomes of adding either liraglutide or rosiglitazone to glimepiride in patients with type 2 diabetes using data from the LEAD-1 clinical trial and a validated simulation model (CORE) of type 2 diabetes.

## Methods

### Background

Data on subject characteristics at baseline and treatment effects were extracted from the LEAD-1 study, which compared the efficacy and safety of three different doses of the once-daily human GLP-1 analog liraglutide (0.6 mg, 1.2 mg and 1.8 mg once daily, OD) added to glimepiride (2–4 mg OD), versus glimepiride alone (placebo) and rosiglitazone (4 mg) in combination with glimepiride, in 1041 type 2 diabetic patients. Patients were stratified based on previous oral antidiabetic drug (OAD) monotherapy or combination therapy and randomly allocated to any of the five arms and followed for 26 weeks. The results of the study showed that all doses of liraglutide plus glimepiride were associated with an improvement in HbA1c and fasting plasma glucose (FPG) levels compared to placebo, and that higher doses of liraglutide (1.2 mg and 1.8 mg) resulted in significantly greater reductions in HbA1c and greater bodyweight loss compared to rosiglitazone. Rates of all hypoglycemic events and nocturnal hypoglycemic events did not significantly differ across treatment arms. For the purposes of this analysis, we focused only on the two highest doses of liraglutide (1.2 mg and 1.8 mg) compared to rosiglitazone, all in combination with glimepiride. The 0.6 mg dose of liraglutide was omitted because it is mainly to be utilized as an escalation dose.

### Model

The CORE Diabetes Model (CDM) has been described in detail previously [31-33]. This interactive computer simulation model has been used to determine the long-term health outcomes and economic consequences of interventions in type 1 or type 2 diabetes using surrogate clinical endpoints, such as HbA1c, systolic blood pressure, lipids, serum cholesterol, and body mass index (BMI) [34-37]. The model has a Markov structure combined with Monte Carlo simulation and the use of tracker variables, which allows for the development and progression of multiple complications in an individual patient over time, improving the limitations of traditional Markov models. The CDM predicts the progression of diabetes type 2 over long-term horizons using the most relevant published epidemiological and clinical data, including studies such as the United Kingdom Prospective Diabetes Study (UKPDS) [38]. The CDM includes 15 sub-models to simulate the most frequent diabetes complications, such as angina, cataracts, congestive heart failure, foot ulcer and amputation, hypoglycemia, ketoacidosis, lactic acidosis, macular edema, myocardial infarction, nephropathy, neuropathy, peripheral vascular disease, retinopathy, stroke, and non-specific mortality. These sub-models run in parallel to allow the hypothetical subjects to develop concomitant complications as appropriate. Cohorts can be defined using demographic characteristics in terms of age, gender, baseline risk factors and pre-existing complications. This model has been validated against 66 published studies, including external (third-order) validation of simulations of type 2 diabetes [32].

### Interventions

Data on the treatment effects of liraglutide 1.2 mg and 1.8 mg or rosiglitazone added to glimepiride were extracted from the LEAD-1 study (Table 1).

**Table 1:** Treatment-specific changes from baseline from LEAD-1 Study

	Liraglutide 1.8 mg		Liraglutide 1.2 mg		Rosiglitazone 4 mg	
	
	Mean change	SD	Mean change	SD	Mean change	SD
HbA1c (%)	-1.13*	1.05	-1.08*	1.04	-0.44	1.05
SBP (mmHg)	-2.81	13.07	-2.56	12.72	-0.93	12.71
Total cholesterol (mg/dl)	-11.99*	37.97	5.06	37.31	7.42	37.14
LDL (mg/dl)	-8.09*	29.85	-2.36	29.28	4.43	29.15
HDL (mg/dl)	-1.57*	7.50	-0.84	7.28	0.75	7.23
Triglycerides (mg/dl)	-14.72*	132.28	-17.64*	130.23	1.73	129.63
BMI	-0.08*	1.11	0.12	1.13	0.78	1.13
Major hypoglycemic event/year	0.01		0		0	
Minor hypoglycemic event/year	0.47		0.50		0.12	

*Statistically significant (p < 0.05) compared to rosiglitazone 4 mg.

BMI, body mass index; HDL, high-density lipoprotein; LDL, low-density lipoprotein; SBP, systolic blood pressure.

### Simulation cohorts

An analytic cohort of 5000 simulated patients was assembled using the treatment-specific baseline demographics and risk factors from the LEAD-1 study (Table 2) [18,25,39,40]. The LEAD-1 study was conducted in 21 countries throughout Europe and Asia. Subjects had a mean duration of diagnosed diabetes of 7.9 years, were 56.1 years old, and had an average BMI of 29.9 kg/m^2^. This trial is described greater in detail by Marre et al. [25]. Treatment specific changes in glycemic control, blood pressure, BMI, and lipids were used to determine the incidence and time to onset of complications, predicted survival, and cost of complications.

**Table 2:** Cohort characteristics at baseline

Characteristic	Baseline value	SD	Reference
Demographics			
Mean age (years)	56.1	9.8	25
Duration of diabetes (years)	7.9	5.4	25
Proportion male (%)	49.4		25
**Risk factors**			
HbA1c (%)	8.4	0.9	25
SBP (mmHg)	132.1	15.4	25
BMI (kg/m^2^)	29.9	5.1	25
Total cholesterol (mg/dl)	196.15	42.3	25
LDL (mg/dl)	130.76	38.46	25
HDL (mg/dl)	50	11.53	25
Triglycerides (mg/dl)	190.9	145.5	25
Ethnic group (%)			
White	64.5		Novo Nordisk, data on file
Black	2.9		Novo Nordisk, data on file
Asian	32.5		Novo Nordisk, data on file
**Cardiovascular disease**			
Stroke (%)	0.9		Novo Nordisk, data on file
Angina pectoris (%)	1.0		Novo Nordisk, data on file
MI (%)	1.4		Novo Nordisk, data on file
CHF (%)	0.1		Novo Nordisk, data on file
Atrial fibrillation (%)	1.5		Novo Nordisk, data on file
LVH by ECG (%)	0.7		Novo Nordisk, data on file
PVD (%)	0.8		Novo Nordisk, data on file
**Renal disease**			
Microalbuminuria (%)	1.1		Novo Nordisk, data on file
Gross proteinuria (%)	0.1		Novo Nordisk, data on file
End-stage renal disease (%)	0.1		Novo Nordisk, data on file
**Retinopathy**			
Background diabetic retinopathy (%)	14.9		Novo Nordisk, data on file
Proliferative diabetic retinopathy (%)	0.1		Novo Nordisk, data on file
**Other complications**			
Peripheral neuropathy (%)	20.0		Novo Nordisk, data on file
Foot ulcer (%)	0.1		Novo Nordisk, data on file
Amputation (%)	0.3		Novo Nordisk, data on file
Cataract (%)	5.6		Novo Nordisk, data on file
Macular edema (%)	0.2		Novo Nordisk, data on file
Severe vision loss (%)	0.1		Novo Nordisk, data on file
**Patient management**			
ACE-I/ARBs (%)	48.7		18
Statins (%)	28.2		18
Aspirin (%)	43.9		18
Screened for retinopathy (%)	67.7		39
Screened for renal disease (%)	55		40
Screened for foot disease (%)	68.3		39

ARB, angiotensin receptor blocker; ACE-I angiotensin-converting enzyme inhibitor; BMI, body mass index; CHF, congestive heart failure; HDL, high-density lipoprotein; LDL, low-density lipoprotein; LVH by ECG, left ventricular hypertrophy confirmed by electrocardiogram; MI, myocardial infarction; PVD, peripheral vascular disease; SBP, systolic blood pressure.

### Analysis

A US healthcare payer perspective was used for the cost analysis. Only direct medical costs of complications are included in the analysis and a discount rate of 3% annually was applied to costs beyond year 1. Table 3 displays the cost inputs used in the simulation [10,41-45]. Drug costs were not applied for the three treatment groups, including the cost of glimepiride, as the price of liraglutide is unknown and applying only the rosiglitazone price would bias the findings in favor of liraglutide. Three analytic time horizons (10, 20 and 30 years) were selected for simulation. Longer-term horizons permit a more complete estimation of complication rates and predicted survival. For sensitivity analysis, the lower and upper limits of the 95% confidence intervals (CI) reported for the changes in HbA1c for each of the three treatment groups were used.

**Table 3:** Cost inputs: US perspective

	Values	Units	Reference
**Discount rates**			
Discount clinical	0.00		41
Discount costs	0.03		41
**Management costs**			
Annual statins	947.74	US$	42
Annual cost aspirin	23.01	US$	42
Annual cost ACE	426.21	US$	42
Annual cost screening for microalbuminuria	18.62	US$	10
Annual cost screening for GFR	27.4	US$	10
Stopping ACEs due to adverse events	0	US$	NA
Annual cost of eye screening	82.18	US$	10
Foot screening program (monthly based)	0	US$	NA
Non-standard ulcer treatment (e.g. topical becaplermin) (monthly based)	167.64	US$	43
**Costs for CVD complications**			
MI year 1	37,421	US$	10
MI year 2 and onwards	2,069	US$	10
Angina year 1	7,424	US$	10
Angina year 2 and onwards	1,917	US$	10
CHF year 1	3,214	US$	10
CHF year 2 and onwards	3,214	US$	10
Stroke year 1	49,556	US$	10
Stroke year 2 and onwards	16,539	US$	10
Stroke death within 30 days	0	US$	NA
PVD year 1	4,707	US$	44
PVD year 2 and onwards	4,707	US$	44
**Costs: renal complications**			
Hemodialysis costs year 1	45,638	US$	10
Annual costs HD year 2 and onwards	45,638	US$	10
Peritoneal dialysis costs year 1	45,638	US$	10
Annual costs PD year 2 and onwards	45,638	US$	10
Renal transplant costs year 1	45,638	US$	10
Annual costs year 2 and onwards	0	US$	NA
**Costs: acute events**			
Major hypoglycemic event	1,191	US$	10
Ketoacidosis event	13,404	US$	10
Lactic acid event	0	US$	NA
**Costs: eye disease**			
Laser treatment	834	US$	10
Cataract operation	2,655	US$	10
Annual costs following cataract operation	0	US$	44
Blindness year 1	4,039	US$	10
Blindness year 2 and onwards	4,039	US$	10
**Costs neuropathy/foot ulcer/amputation**			
Neuropathy year 1	408	US$	10
Neuropathy year 2 and onwards	408	US$	10
Amputation (event-based)	33,257	US$	10
Amputation prosthesis (event based)	1,195	US$	10
Gangrene treatment (monthly based)	6,240	US$	45
After healed ulcer (yearly based)	0	US$	NA
Infected ulcer (monthly based)	3,198	US$	45
Standard uninfected ulcer (monthly based)	1,769	US$	45
Healed ulcer history of amputation (yearly based)	0	US$	NA

ACE, angiotensin-converting enzyme; CHF, congestive heart failure; CVD, cardiovascular disease; GFR, glomerular filtration rate; MI, myocardial infarction; PVD, peripheral vascular disease.

## Results

Table 4 reports the predicted survival, cardiovascular mortality, event rates for complications and costs. These results are reported for the three treatment groups in LEAD-1 and for the three analytic time horizons.

**Table 4:** Predicted survival, events and costs by treatment group and time horizon

Treatment group	Time horizon (years)	Survival rates (%)	Number of events in a hypothetical population of 5000 subjects								Average cumulative costs of complications per patient (US$- discounted)
				
			CVD	MI	Stroke	CHF	Renal disorders (including ESRD death)*	Visual disorders†	Amputation (first and recurrent)		
			N (%)								
Liraglutide 1.2 mg	10	82.4	727	14.54%	346	140	381	621	1261	144	14,126.53
	20	49.0	2,049	40.98%	900	381	992	1322	2534	384	29,850.63
	30	15.0	3,484	69.68%	1373	563	1476	1756	3242	565	38,963.07
Liraglutide 1.8 mg	10	82.3	728	14.56%	355	160	391	622	1271	115	14,162.06
	20	49.2	2,017	40.34%	881	421	987	1296	2578	358	30,021.86
	30	16.0	3,419	68.38%	1323	611	1478	1695	3233	529	39,239.92
Rosiglitazone 4 mg	10	80.8	782	15.64%	444	161	422	804	1548	113	15,237.10
	20	45.5	2,227	44.54%	1062	385	1060	1541	2910	347	31,243.92
	30	12.6	3,624	72.48%	1574	586	1489	1923	3529	507	40,401.96

*Microalbuminuria + Gross proteinuria + ESRD + ESRD death.

^†^Background retinopathy + proliferative retinopathy + macular edema + severe vision loss + Cataract.

CHF, congestive heart failure; CVD, cardiovascular death; ESRD, end-stage renal disease MI, myocardial infarction.

As expected, predicted overall survival declined and complication rates increased for all three treatments as the analytic horizon was extended from 10 to 30 years. Overall survival in both liraglutide-treated groups was higher than in the rosiglitazone-treated group at all three time points. After 30 years the differences in survival were 2.4% and 3.6% higher in the group treated with liraglutide 1.2 mg and 1.8 mg respectively, compared to rosiglitazone. Complication rates were higher at all three time points for the rosiglitazone group compared to the two liraglutide groups.

**Figure 1:**
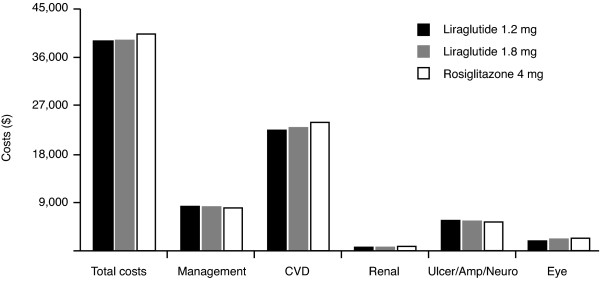
**Breakdown of medical costs. CVD, cardiovascular disease**.

Applying the unit cost data in Table 3 to the event rate predictions from Table 4 produced an estimate of total costs of complications during the follow-up up to 30 years excluding the costs of liraglutide and rosiglitazone as the cost for the former is presently unknown since the medication is not presently FDA approved or marketed. Total cumulative costs per patient, defined as the management costs, costs of ongoing disease complications and costs of acute events related to the disease, during the 30 years of follow-up were $276 dollars lower in the group treated with liraglutide 1.2 mg compared to liraglutide 1.8 mg, and $1438 dollars lower compared to the rosiglitazone group (Table 4). As expected, the costs related to cardiovascular events were the main factor in all groups, representing 57.4% of the total costs per patient for liraglutide 1.2 mg, 58.5% for liraglutide 1.8 mg, and 59.1% for rosiglitazone. Management costs and costs related to the treatment of ulcers, amputations and neuropathies were lower in the rosiglitazone group (Figure 1).

We used the upper and lower limits of the 95% CI of the reported changes in HbA1c for each of the three treatment groups to evaluate the sensitivity of our findings to uncertainty in the treatment benefit. The absolute survival and event rates changed slightly across all three time periods: less than 5% in either direction depending on whether the simulations were run using the upper or lower bound of the CI. In neither case did the model produce predicted outcomes for the rosiglitazone group that were better than either liraglutide group.

## Discussion

As there are no long-term follow-up studies of liraglutide or rosiglitazone measuring mortality as the primary endpoint, reliance must be placed on simulation models that have reproduced accurately the outcomes of long-term cohorts of patients with diabetes [46]. Our modelling study has shown that, in patients with type 2 diabetes treated with glimepiride, adding liraglutide 1.8 mg or 1.2 mg, compared to adding rosiglitazone 4 mg, may lead to improved survival and reductions in complications over a 10 to 30-year period. Additionally, the groups treated with liraglutide had a higher projected survival rate and lower cumulative medical costs, compared to rosiglitazone. These differences increase over time but are noticeable even after the first 10 years of follow-up. The lower number of complications related to ulcers and amputations in the rosiglitazone treatment group, compared to the two groups with liraglutide, may be explained in part by the lower survival time, as there is less chance of this type of complication with the shorter exposure time.

Other events, such as visual disorders, are influenced by additional factors, especially changes in blood pressure, hence the effects of the therapies on blood pressure should be considered and could support an explanation of these differences; in the LEAD-1 study, liraglutide 1.8 mg and 1.2 mg showed a higher reduction of systolic blood pressure compared to rosiglitazone (-2.81 mmHg, -2.56 mmHg, and -0.93 mmHg, respectively).

As expected, cardiovascular events were the leading cause of death across all groups; nevertheless the survival rate was relatively higher than that usually expected in these patients. This may be caused by a study effect, as the population from the LEAD-1 study, used for this simulation study, could be considered 'healthier' than the average type 2 diabetic patient after 8 years of diagnosis. Most patients were recruited in European and Asian countries, with mean near normal levels of total cholesterol, low-density lipoprotein, high-density lipoprotein, and triglycerides. Further, a very low proportion of patients reported a previous cardiovascular event or renal impairment at baseline.

Cardiovascular disease was also the main contributor to the cumulative costs for all groups, and explains in great part the overall higher costs of the rosiglitazone group, despite the lower survival time of this group, and lower costs in management and complications related to neuropathy, ulcers, and amputations. The safety of liraglutide and rosiglitazone, specifically regarding minor and major hypoglycemic events, was also projected from the LEAD-1 study, probably underestimating the real effect in events and costs for a longer follow-up. Treatment switching, dose adjustments and adherence were not considered to have any effect in the simulation, thus providing an ideal scenario that may be more optimistic than actual practice, but the effect could be non-differential across treatments and therefore keep the trend in differences as reported here, probably with a higher number of events earlier in the follow-up.

We would like to point out a few additional limitations of the research. Although the model uses data from epidemiologic and clinical trials, some recently published studies have called into question the cardiovascular benefits of intensive glycemic control in patients with longer duration of diabetes and/or existing diabetes complications [18,22]. It is important to note that, at the time of writing, these newer data have not yet been incorporated into the CORE diabetes model, so the potentially negative effect of more intensive treatments is not considered; only information from the ADVANCE trial was used as the reference for the current management of diabetic patients in the use of aspirin, statins, and angiotensin receptor blocker (ARB)/angiotensin-converting enzyme (ACE) inhibitors. The effect of rosiglitzaone on systolic blood pressure in LEAD-1 may be underestimated when compared to evidence from other trials [47]. One important caution when interpreting the results of the cumulative costs is that the costs of adding liraglutide or rosiglitazone to glimepiride treatment are not included because liraglutide is not on the market and the price is not known. More specific research will be required to determine the cost-effectiveness of the treatments.

In the sensitivity analysis, only changes in HbA1c were considered, using the lower and upper limits of the 95% CI for every treatment as this was the primary endpoint of the LEAD-1 study. Nevertheless, other significant changes in the study, that is, blood pressure, lipids and weight, could have been included, thereby increasing the uncertainty of estimates in the simulation but assessing a more comprehensive effect of these therapies.

Finally, the utility of diabetes models to predict life expectancy and other disease outcomes with precision is open to criticism. Models are imperfect instruments of real world outcomes. Nevertheless, attempts to correlate diabetes model predictions of outcomes with the results of long-term trials have been undertaken. These studies have shown that models can produce findings broadly consistent with long-trials under specified conditions [48].

## Conclusion

This study represents one of the first uses of a disease simulation model to examine the long-term clinical effects of a GLP-1 by incorporating data from a head-to-head active comparator clinical trial. This study represents an important advance relative to previously published works, which were based on modelling data from placebo-controlled clinical trials [39]. Notably, the availability of head-to-head clinical trial data and the incorporation of active-comparator designs as part of the registration study program provide valuable additional therapeutic information for healthcare decision-makers during the immediate post-launch experience.

This study shows that in patients with type 2 diabetes treated with glimepiride, adding liraglutide 1.8 mg or 1.2 mg, compared to adding rosiglitazone 4 mg, may improve survival rates and reductions in complications over a 10- to 30-year period. The liraglutide 1.2 mg and 1.8 mg groups had higher projected survival rates and lower cumulative costs, compared to rosiglitazone 4 mg.

Improvements in the cardiovascular event rates are important as these events are the main contributor to death and increased cost of treating type 2 diabetes.

## Competing interests

SDS has received research support from Novo Nordisk. He has received consulting fees from Novo Nordisk, Amylin and Novartis. RA-C worked for Merck Sharp & Dohme, Colombia to 2004, then for sanofi-aventis Latin America (Panama), until August 2007, and has acted as a consultant for sanofi-aventis Latin America thereafter. CC and MH are employees of Novo Nordisk. LB has acted as a consultant for, has attended speakers' bureaux for, and is a shareholder in Amylin Pharmaceuticals, Inc., is a share holder in and has attended speakers bureaux for Eli Lilly & Co.

## Authors' contributions

All authors contributed equally to the design, analysis and interpretation of study findings. However, decisions regarding final analysis and interpretation of findings were the responsibility of the first author, SDS. All authors contributed to the writing process and approved the final manuscript.

## Authors' information

SDS and ACR are health economic researchers based at the University of Washington, Seattle, USA. CC work as senior health economist in Novo Nordisk Inc, USA. MH is principal scientist in health economic of Novo Nordisk A/S, Denmark. LB is a diabetologist working in clinical practice in USA.
